# Deep Learning on Enhanced CT Images Can Predict the Muscular Invasiveness of Bladder Cancer

**DOI:** 10.3389/fonc.2021.654685

**Published:** 2021-06-11

**Authors:** Gumuyang Zhang, Zhe Wu, Lili Xu, Xiaoxiao Zhang, Daming Zhang, Li Mao, Xiuli Li, Yu Xiao, Jun Guo, Zhigang Ji, Hao Sun, Zhengyu Jin

**Affiliations:** ^1^ Department of Radiology, Peking Union Medical College Hospital, Peking Union Medical College and Chinese Academy of Medical Sciences, Beijing, China; ^2^ Department of Radiology, Fushun Central Hospital of Liaoning Province, Fushun, China; ^3^ Deepwise Artificial Intelligence (AI) Lab, Deepwise Inc., Beijing, China; ^4^ Department of Pathology, Peking Union Medical College Hospital, Peking Union Medical College and Chinese Academy of Medical Sciences, Beijing, China; ^5^ Department of Urology, Peking Union Medical College Hospital, Peking Union Medical College and Chinese Academy of Medical Sciences, Beijing, China

**Keywords:** bladder cancer, deep learning, computed tomography, diagnosis, computed-assisted, artificial intelligence

## Abstract

**Background:**

Clinical treatment decision making of bladder cancer (BCa) relies on the absence or presence of muscle invasion and tumor staging. Deep learning (DL) is a novel technique in image analysis, but its potential for evaluating the muscular invasiveness of bladder cancer remains unclear. The purpose of this study was to develop and validate a DL model based on computed tomography (CT) images for prediction of muscle-invasive status of BCa.

**Methods:**

A total of 441 BCa patients were retrospectively enrolled from two centers and were divided into development (n=183), tuning (n=110), internal validation (n=73) and external validation (n=75) cohorts. The model was built based on nephrographic phase images of preoperative CT urography. Receiver operating characteristic (ROC) curves were performed and the area under the ROC curve (AUC) for discrimination between muscle-invasive BCa and non-muscle-invasive BCa was calculated. The performance of the model was evaluated and compared with that of the subjective assessment by two radiologists.

**Results:**

The DL model exhibited relatively good performance in all cohorts [AUC: 0.861 in the internal validation cohort, 0.791 in the external validation cohort] and outperformed the two radiologists. The model yielded a sensitivity of 0.733, a specificity of 0.810 in the internal validation cohort and a sensitivity of 0.710 and a specificity of 0.773 in the external validation cohort.

**Conclusion:**

The proposed DL model based on CT images exhibited relatively good prediction ability of muscle-invasive status of BCa preoperatively, which may improve individual treatment of BCa.

## Introduction

Bladder cancer (BCa) is one of the most common and lethal malignancies worldwide (1, 2). Clinical treatment decision making primarily relies on the absence or presence of muscle invasion and tumor staging (3). Nonmuscle-invasive BCa (NMIBC) and muscle-invasive BCa (MIBC) exhibit significant differences in prognosis, management and therapeutic aims (3, 4). Accurate preoperative assessment of the muscular invasiveness of BCa is crucial for selecting the optimal therapy for individual patients.

Cystoscopy examination together with histological evaluation of the resected tissues is the mainstay of diagnosis and clinical staging of BCa. As biopsy is operator dependent and unlikely to sample every part of the tumor, incorrect staging occurs, and up to 25% of MIBC cases are initially misdiagnosed as NIMBC (5, 6). Repeated examinations could improve the diagnostic accuracy, but the invasive nature has made this process undesirable. Developing a noninvasive method for preoperative evaluation would greatly benefit BCa patients. Computed tomography (CT) imaging has been widely used to preoperatively evaluate BCa patients and assist in tumor staging, especially for T3 and T4 tumors (7). Given its inability to differentiate among layers of the bladder wall, the role of traditional CT in the classification of NIMBC and MIBC is limited. Thus, developing a technique that could provide additional information about the status of muscular invasion of BCa would enable traditional CT to play a larger role in BCa evaluation and assist in patient management.

Deep learning (DL) is a novel and promising technique that has demonstrated great potential in disease diagnosis (8–10). DL can extract and combine features from images to construct a model that reveals the relationship between images and diseases. It has been reported that the DL model could facilitate imaging diagnosis in various diseases with high accuracy, including liver fibrosis, pancreatic cancer and pulmonary nodules (9, 11, 12). For BCa, the DL model based on CT images has demonstrated the potential to assist in therapy evaluation (13). However, the use of the DL based on CT images to discriminate between MIBC and NIBC has not yet been reported.

Therefore, the aim of this study was to develop and validate a DL model based on CT images for individualized prediction of the muscle-invasive status of BCa preoperatively.

## Materials and Methods

### Study Population

**Figure 1:**
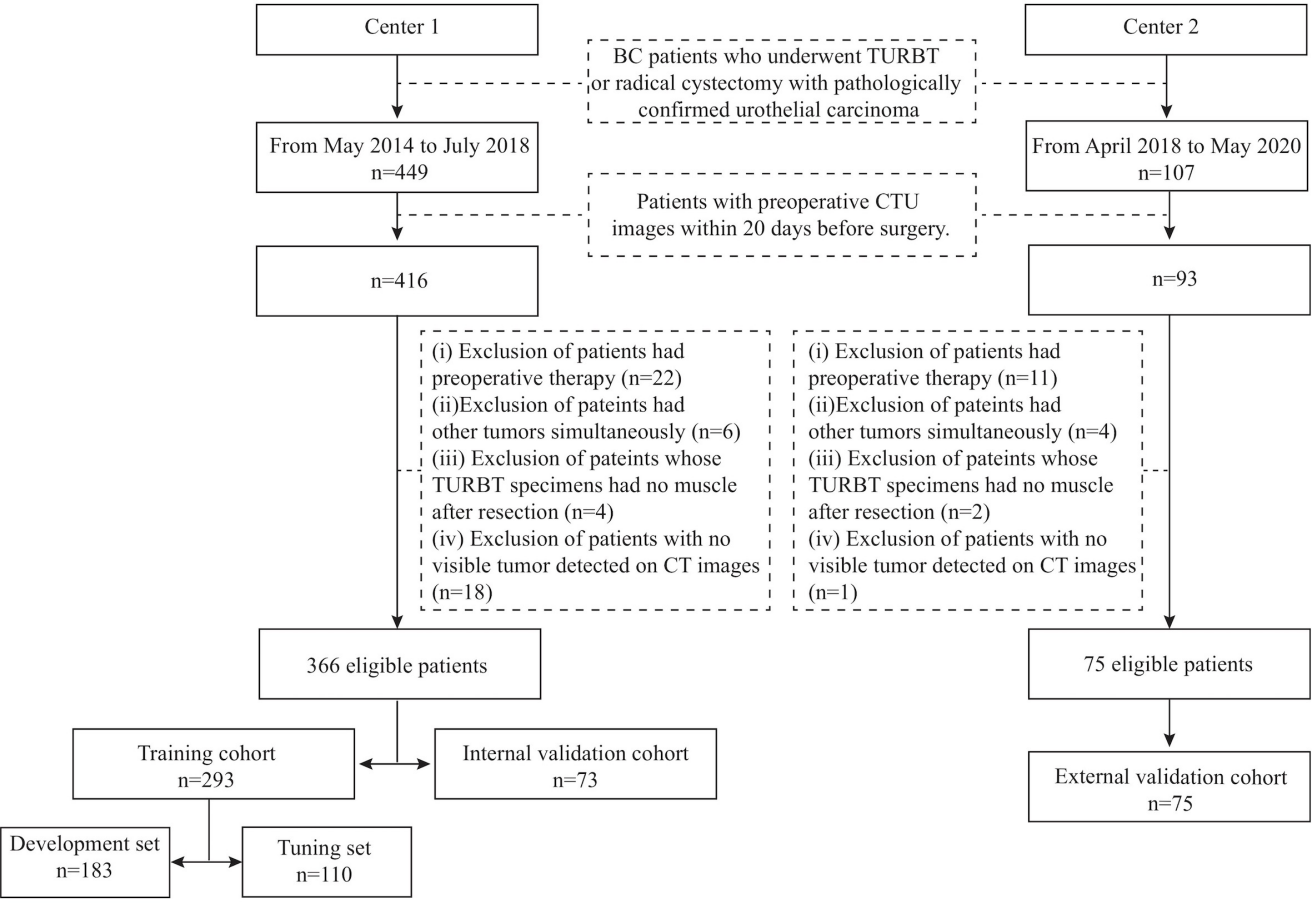
The study flow and the recruitment pathway. BC, bladder cancer; TURBT, transurethral resection of bladder tumor; CTU, computed tomography urography.

This retrospective study was approved by the Institutional Review Board of the two medical centers, and the requirement of informed consent was waived. The inclusion criteria were as follows: (i) patients who underwent transurethral resection of bladder tumor (TURBT) or radical cystectomy in the two centers with pathologically confirmed urothelial carcinoma and (ii) availability of preoperative CT urography (CTU) within 20 days before surgery. Patients were excluded if (i) they had preoperative therapy, including chemotherapy or radiotherapy; (ii) they had other tumors simultaneously; (iii) their TURBT specimens had no muscle after resection; or (iv) no visible tumor was detected on preoperative enhanced pelvic CT images. Two radiologists (H.S. in Center 1 and Z.W. in Center 2) identified patients according to the above criteria, and 366 patients were recruited from May 2014 to July 2018 from Center 1 (91 patients with MIBC, 275 patients with NIMBC) and 75 patients from April 2018 to May 2020 in Center 2 (31 patients with MIBC, 44 patients with NIMBC). We divided the patients into three cohorts: 293 patients treated between May 2014 and September 2017 in Center 1 were allocated to the training cohort, 73 patients treated between October 2017 and July 2018 in Center 1 were allocated to the internal validation cohort, and all 75 patients treated in Center 2 constituted the external cohort. The training cohort was further randomly assigned into a development set (n=183) for model training and a tuning set (n=110) for model selection. The study flow and recruitment pathway are presented in **Figure 1**.

Clinical-pathologic information, including age, sex and pathologic T stage, was obtained from medical records. Two experienced radiologists (6 and 14 years of experience in in urogenital imaging) reviewed all the CT images together and recorded data, including the number of tumors, the size and the CT attenuation of the largest tumor. Any disagreement was resolved by consensus.

### CT Imaging

All the enrolled patients in both centers underwent preoperative CTU with a similar protocol setup with different systems. The CT image acquisition settings are provided in **Supplementary Table S1**. Patients fasted for 4-6 hours, and then were asked to drink about 1000 ml water about 45 minutes before the scan and not to urinate until the scan was finished. Patients were scanned from the hemidiaphragm to the pelvic floor. For the contrast scans, patients were injected with 100 ml of nonionic contrast material (Ultravist 370, Bayer Schering Pharma AG, Germany) followed by a 100-ml saline chaser intravenously at a rate of 4–4.5 mL/s after the unenhanced scan. Renal corticomedullary-phase, nephrographic-phase and excretory-phase images were acquired at 25 s, 75 s and 300 s after the bolus-triggering threshold of 120 HU was achieved in the thoracoabdominal aorta junction. To show BCa lesions better, coronal and sagittal reformations were reconstructed besides axial images. But only the axial nephrographic-phase images were used for subsequent analysis.

### Tumor Region Segmentation

Regions of interest (ROIs) were delineated semiautomatically on thin-slice CT images of the nephrographic phase by an experienced radiologist (G.Z., 6 years of experience in urogenital imaging and 5 years of experience in tumor segmentation) who was blinded to the pathological status of muscular invasion of lesions. For patients with multiple lesions, only the largest lesion was chosen for segmentation. A three-dimensional ROI of the whole tumor was delineated semiautomatically using the Deepwise Research Platform (Deepwise Inc., Beijing, China, http://label.deepwise.com). On the platform, a level-set-based segmentation algorithm was initially used to outline the tumor margin automatically, and then the radiologist manually corrected the tumor margin where it was not accurate. After 8 weeks, 93 patients in the development set were selected randomly, and their tumors were segmented again by the same radiologist and another radiologist (X.Z., 1 year of experience in urogenital imaging and tumor segmentation) to evaluate intra- and interobserver reproducibility by calculating intra- and interclass dice coefficients.

### Development and Validation of the Model

**Figure 2:**
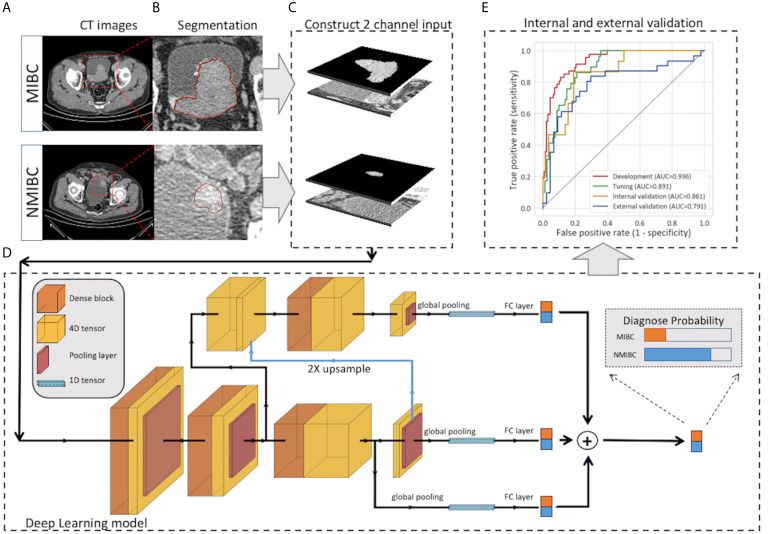
Workflow of the deep learning model for the prediction of muscle invasiveness status in bladder cancer patients. **(A)** Collection of the CT images of MIBC and NMIBC. **(B)** Semiautomatic segmentation of the tumor region. **(C)** The masked tumor region and the original tumor region were stacked vertically to form the input volume, and the cropped 2-channel input was constructed. **(D)** The structure of our deep-learning model. The model was constructed on the basis of Filter-guided Pyramid Network (FGP-Net), a novel 3D convolutional network structure that is designed to capture the global feature and the local features simultaneously. **(E)** Internal and external validation of our model. CT, computed tomography; FC, fully connected layer.

The pipeline of DL modeling is presented in **Figure 2**. Before the training of the model, the images were preprocessed. The voxel size was normalized to 1.0 x 1.0 x 1.0 mm3, and the pixel values were rescaled to (0,1). To further utilize the segmentation and focus the model’s attention on the tumor area, the masked tumor region and the original tumor region were stacked vertically, then cropped it according to the tumor center to form an input volume of 2 x 64 x 64 x 64 for channel, depth, height and width, respectively. Our model was constructed on the basis of Filter-guided Pyramid Network (FGP-Net), a novel 3D convolutional network structure that was designed to capture the global feature and the local features simultaneously in our previous study (14). To avoid overfitting, the growth rate of the dense block was reduced to 8, and a dropout layer with a drop rate of 0.5 was added. In addition, the input patches were augmented by random cropping and rotation during the training process. The output of our model was the probability of the MIBC. Focal loss with a gamma of 1.5 and a class weight of 3 were used to manage the unbalanced amount of MIBC and NMIBC tumors. The Adam optimizer was used to minimize the focal loss with an initial learning rate of 0.001 (15). The output of our model was the probability of the MIBC, the model that achieved the highest area under the receiver operating characteristic curve (AUC) on the tuning set during the training procedure was selected, and the cut-off value was selected at the points that maximized the Youden index value on the tuning set. The AUC, accuracy, sensitivity, and specificity of all sets were calculated. The calibration curve with LOESS smoother was generated to assess the calibration of the DL model (16).

Two methods that visualizing the feature extraction process by the convolutional neural network were used to demonstrate whether the DL model learned valuable features from meaningful CT areas. First, the feature maps before discriminative filter learning modules in our model were extracted to show the target area of the model. The value of the area on the feature map indicated its contribution to the final result. The higher the value, the larger the contribution of the area. Using gamma correction (γ=2.0), the feature maps were transformed, mapped to a colored scheme and overlaid on the original images. Second, t-distributed stochastic neighborhood embedding (t-SNE), which is an unsupervised dimension-reduction algorithm to visualize high-dimensional data, was used to test the effectiveness of the learned features. In this study, t-SNE was used to reduce the dimension of features (the output of the layer before the final fully connected layer) from 150 to 2 with a learning rate of 450 and a perplexity of 30.

### Subjective Image Evaluation

For subjective assessment of muscular invasion of BC based on CT images, a tumor was defined as MIBC if it invaded perivesical fat with the tumor bulging out or based on the presence of abnormal enhancement of bladder wall; otherwise, it was considered NIMBC. Examples of these imaging features were demonstrated to two radiologists (Reader 1, L.X., Reader 2, D.Z., with 3 and 9 years of experience in CTU, respectively) before they started the review process. The two radiologists reviewed all the images in validation cohorts (n=148) and determined whether the tumor was MIBC or NMIBC independently, without knowledge of pathological information (including the status of muscular invasiveness of tumors). For patients with multiple tumors, only the largest tumor was evaluated. The performance of the two radiologists for diagnosing MIBC was evaluated by calculating accuracy, sensitivity and specificity.

### Statistical Analysis

A two-sided *P*<0.05 indicated statistically significant differences. Analysis of variance or Kruskal-Wallis H test was used to compare clinical characteristics among development, tuning, internal and external validation cohorts. These statistical analyses were performed by using SPSS version 25.0 (IBM, SPSS; Chicago, IL, USA). The comparison of the AUC was calculated by the DeLong test (17) which was performed by using R (version 3.6.0). The ROC curves, decision curve analysis (DCA) and calibration curves were calculated using scikit-learn (version 0.22.1) and matplotlib (version 3.1.3).

## Results

### Patient Clinical Characteristics

Patient characteristics in all the cohorts are shown in **Table 1**. No significant differences in gender or CT-reported largest lesion diameter (*P* > 0.05) were noted among the training, internal validation and external validation cohorts. Patient age, CT-reported number of lesions, CT attenuation of the largest lesion and pT stage were significantly different. The proportion of MIBC was significantly increased in the external validation cohort (*P* = 0.010).

**Table 1:** Clinical characteristics of patients with bladder cancer.

Characteristics	Training cohort^*^(n=293)	Internal validation cohort (n=73)	External validation cohort (n=75)	p-value
Age				0.038
Median (IQR)	65 (56,72)	68 (61,74)	65 (59,77)	
Gender				0.166
Female	75 (25.6)	13 (17.8)	13 (17.3)	
Male	218 (74.4)	60 (82.2)	62 (82.7)	
CT-reported number of lesions				0.016
Unifocal	229 (78.2)	66 (90.4)	54 (72.0)	
Multifocal	64 (21.8)	7(9.6)	21 (28.0)	
CT-reported largest lesion diameter (cm)				0.063
Mean ± SD	2.71 ± 1.67	2.33 ± 1.62	2.78 ± 1.70	
≤3	188 (64.2)	57 (78.1)	52 (69.3)	
>3	105 (35.8)	16 (21.9)	23 (30.7)	
CT attenuation of the largest lesion (HU)				0.030
Mean ± SD	67.1 ± 14.0	56.3 ± 20.9	70.5 ± 13.0	
Pathologic T stage				0.010
≤T1	217 (74.1)	58 (79.5)	44 (58.7)	
≥T2	76 (25.9)	15 (20.5)	31 (41.3)	

^*^The training cohort (n=293) is the combination of the development (n=183) and tuning (n=110) cohorts. IQR, interquartile; SD, standard deviation.

### The Performance Assessment and the Clinical Usefulness of the Model

**Figure 3:**
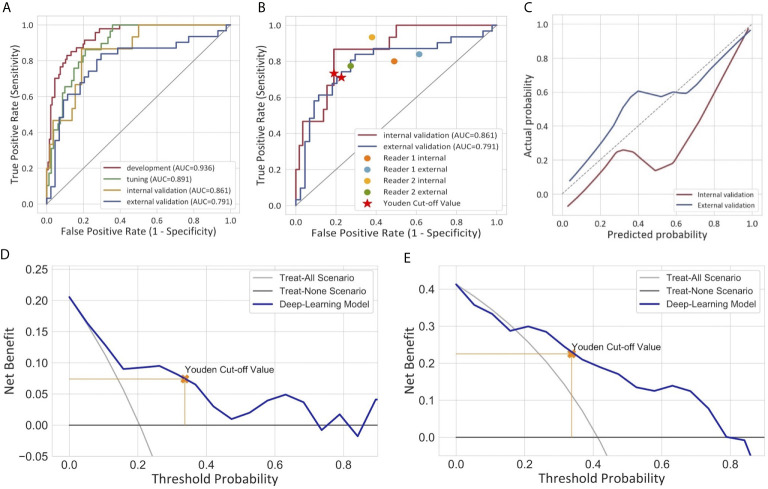
Performance of the deep learning model for the differentiation of MIBC and NMIBC. **(A)** Receiver operator characteristic curves of the model in four different cohorts. **(B)** Comparison of the performance between the model and two radiologists. **(C)** Calibration curves of the model in internal and external validation cohorts. The calibration curve showed that the predicted probabilities generally agreed with the observed probabilities. The predictive performance of the model in the external validation cohort exhibited a closer fit to the perfect calibration. **(D, E)** showed decision curve analyses (DCA) in the internal and external validation cohorts respectively. DCA compared the net benefit of the deep learning model versus treat all or treat none are shown. The net benefit was plotted versus the threshold probability. The net benefits of the deep learning model (blue line) were superior to the benefits of treating all or treating none.

For the semiautomatic segmented ROI, the intraclass dice coefficient (0.800 ± 0.201) indicating favorable reproducibility, while the interclass dice was relatively low (0.706 ± 0.253). The ROC curves of the DL model are presented in **Figure 3A**. The model produced satisfactory performance in the development (AUC 0.936) and tuning (AUC 0.891) cohorts. The AUC in the internal validation cohort and the external validation cohort reached 0.861 (95% CI: 0.765, 0.957) and 0.791 (95% CI: 0.678, 0.904), respectively, demonstrating good differentiating ability between MIBC and NMIBC and good model robustness. The cut-off value that maximized the Youden index was 0.337. The performance of our model for differentiating between MIBC and NMIBC on development and tuning sets is also summarized in **Table 2**.

**Table 2:** Performance of the model in development, tuning and validation cohorts.

	AUC (95%CI)	Accuracy (95%CI)	Sensitivity (95%CI)	Specificity (95%CI)
Development cohort (n=183)	0.936 (0.901, 0.971)	0.836 (0.773, 0.885)	0.872 (0.736, 0.947)	0.824 (0.747, 0.882)
Tuning cohort (n=110)	0.891 (0.832, 0.950)	0.800 (0.711, 0.868)	0.828 (0.635, 0.935)	0.790 (0.683, 0.87)
Internal validation cohort (n=73)	0.861 (0.765, 0.957)	0.795 (0.681, 0.877)	0.733 (0.448, 0.911)	0.810 (0.682, 0.897)
External validation cohort (n=75)	0.791 (0.678, 0.904)	0.747 (0.631, 0.837)	0.710 (0.518, 0.851)	0.773 (0.618, 0.880)

AUC, area under the receiver operating characteristics curve; CI, confidence interval.

The calibration curves of the model exhibited good agreement between the model predicted outcome and the real status of muscular invasiveness (**Figure 3C**). The DCA indicated that the DL model could add more benefit to patients than the “treat all” or “treat none” strategies when the threshold probability was ranged from 0 to 0.74 in the internal validation cohort and 0.21 to 0.79 in the external validation cohort (**Figures 3D, E**).

### The Comparison With Radiologists

In the subjective assessment of muscular invasion of BCa, the two radiologists generally performed slightly worse compared with the DL model (**Table 3** and **Figure 3B**). In the internal validation cohort, the accuracy and specificity of Reader 1 (0.685 and 0.621) and Reader 2 (0.585 and 0.517) were lower than those of the model (0.795 and 0.810), while the sensitivity of them (0.933 and 0.800) exceeded that of the model (0.733). In the external validation cohort, Reader 1 demonstrated comparable performance compared to the model with the same accuracy (0.747) and similar specificity (0.727 *vs* 0.773) and sensitivity (0.774 *vs* 0.710). However, the performance of Reader 2 was inferior to the model in general with a lower accuracy (0.573 *vs* 0.747) and specificity (0.386 *vs* 0.773) but higher sensitivity (0.839 *vs* 0.710).

**Table 3:** Performance of two radiologists and the deep learning model on validation cohorts.

Validation cohort	Reader	Accuracy(95%CI)	Sensitivity(95%CI)	Specificity(95%CI)
Internal	Reader 1	0.685 (0.564, 0.786)	0.933 (0.660, 0,.997)	0.621 (0.483, 0.742)
	Reader 2	0.585 (0.454, 0.688)	0.800 (0.514, 0.947)	0.517 (0.383, 0.649)
	Model	0.795 (0.681, 0.877)	0.733 (0.448, 0.911)	0.810 (0.682, 0.897)
	Reader 1	0.747 (0.631, 0.837)	0.774 (0.585, 0.897)	0.727 (0.570, 0.845)
External	Reader 2	0.573 (0.454, 0.685)	0.839 (0.655, 0.939)	0.386 (0.247, 0.545)
	Model	0.747 (0.631, 0.837)	0.710 (0.518, 0.851)	0.773 (0.618, 0.880)

AUC, area under the receiver operating characteristics curve; CI, confidence interval.

### Additional Analysis

**Figure 4:**
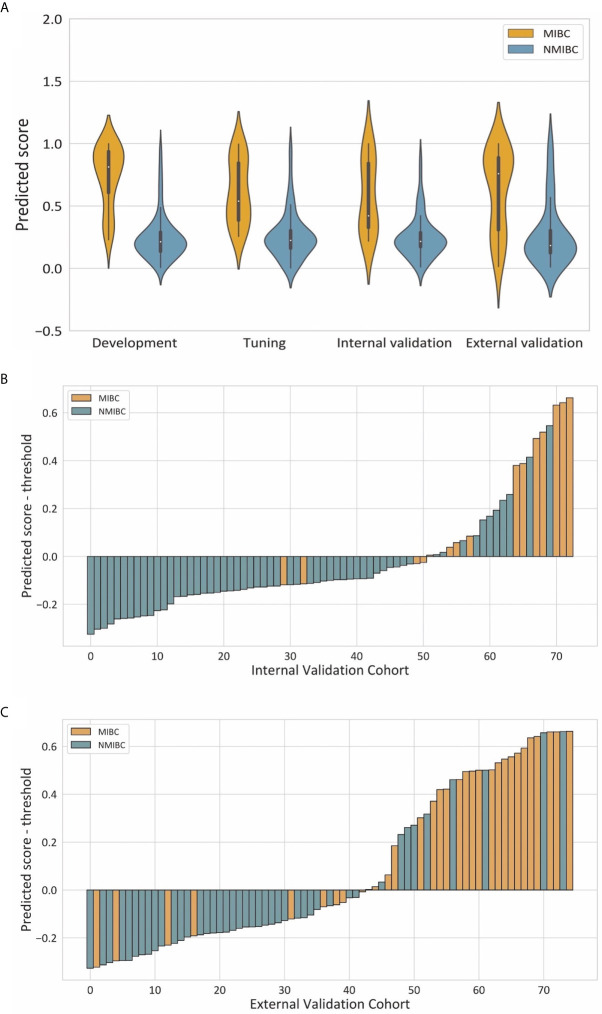
Illustrations of the performance of the deep learning model. **(A)** Violin plots of predictive scores in the development, tuning, internal validation and external validation cohorts. **(B, C)** showed waterfall plots of the distribution of predictive scores and muscle invasive status of each patient in the internal and external validation cohorts respectively.

Violin plots of the predicted score for muscle invasion in the development, tuning, internal and external cohorts are shown in **Figure 4A**. NMIBC patients had significantly lower predicted scores than those with MIBC in the development (median 0.214 [interquartile range 0.136-0.291] *vs* 0.813 [0.607, 0.938], *P<* 0.001), tuning (0.225 [0.161, 0.304] *vs* 0.539 [0.385, 0.846], *P* < 0.001), internal validation (0.216 [0.172, 0.288] *vs* 0.422 [0.327, 0.843], *P* < 0.001) and external validation (0.184, [0.124, 0.305] *vs* 0.759 [0.307, 0.889], *P* < 0.001) cohorts. The waterfall plots in **Figures 4B, C** illustrate the distribution of the predicted score and the status of muscular invasion of individual patients in the internal and external validation cohorts, respectively.

**Figure 5:**
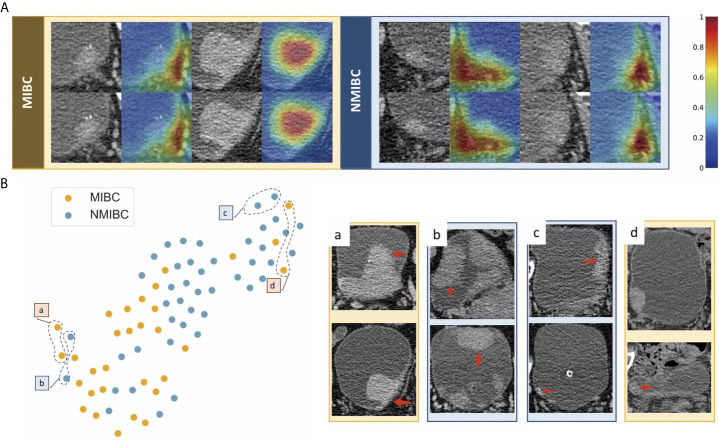
Examples of feature maps from validation cohorts and visualization of the effectiveness of the learned features. **(A)** Two cases from MIBC and two cases from NMIBC are shown. The active regions were mainly overlaid on the areas with visual characteristics that were helpful for discriminating between MIBC and NMIBC, including the internal region of the tumor, corresponding bladder wall, and the surrounding outside pelvic fat. **(B)** Colored points represent the NIMBC (blue) and MIBC (orange). Effective features were learned by the model, and the two categories of nodules were well clustered. The eight examples show images corresponding to circled points. Nodules in sets a and c were highly discriminated by the model, whereas nodules in sets b and d were less discriminated because they shared similar features with the opposite tumor.

We used feature maps and t-SNE to visualize the learned features. **Figure 5A** demonstrates feature maps of four examples (two for MIBC and two for NMIBC) from the external validation cohort. The focus area of the model or the active area is illustrated by bright colors. These regions represent different characteristics of lesions and were in accord with human observations, and the models would aid in the classification of lesions. T-SNE visualization demonstrated that the learned features of the DL model can distinguish MIBC and NMIBC. The locations of BC lesions depended on the similarity of their features. They were close to each other if they had similar features; otherwise, they were far apart. As shown in **Figure 5B**, MIBC and NMIBC clusters were basically separated except for several outliners, demonstrating that the developed model has captured effective features for differentiation.

## Discussion

The aim of this double-center study was to predict the muscular invasiveness of bladder cancer based on enhanced CT images. Our DL model exhibited relatively good performance to discriminate NMIBC from MIBC. The AUC was 0.861 in the internal validation cohort and 0.791 in the external validation cohort.

Preoperative evaluation of muscle invasion in bladder cancer is important for patient management. Currently, transurethral resection of bladder tumor is the standard for preoperative T staging evaluation (3, 7, 18–21). As the procedure highly depends on surgeon experience and biopsy quality, its diagnostic accuracy for MIBCs varies. MRI is also recommended, and the Vesical Imaging-Reporting and Data System based on multiparametric MRI has been proposed for the diagnosis of MIBCs (22). But it is still a subjective evaluation process based on the experience of radiologists. In recent years, researchers have investigated alternative techniques to assist muscle invasiveness evaluation. Garapati et al. (23) explored machine learning methods to discriminate between MIBC and NMIBC in 84 BC lesions from 76 CTU cases retrospectively. They found that morphological and texture features achieved comparable performance with AUCs of about 0.90. Some other studies developed MRI-based radiomic models for preoperative prediction of the muscle-invasive status of BCa with AUCs ranging from 0.87 to 0.98 (24–27). These studies revealed encouraging results for avoiding subjectivity in the preoperative assessment of BCa, but external validation in larger cohorts is required to verify the clinical validity of these new techniques. In contrast to the above studies, we investigated the feasibility of using DL on CT images to differentiate between MIBC and NMIBC. We used a well-designed deep learning structure, which utilizes the dense block and the pyramid structure to extract the features effectively and integrate the global features and the local features (14). Considering the relatively small sample size, several methods were utilized to alleviate the problem of overfitting, including reducing the growth rate of the dense block and data augmentation. The focal loss, which is designed to handle the imbalance of the data amount and the difficulty, was employed (15). Regarding diagnostic performance, the AUCs in this study were slightly lower than those in other studies. When we analyzed what went wrong and why, we found that most true NMIBC cases that were mistakenly identified as MIBC were large (typically >4 cm), and almost all the MIBC cases falsely recognized as NIMBC were small (typically < 1 cm). These findings suggest that the DL model considers the tumor size as one of the key features to determine the muscle-invasive status of BCa.

In general, the DL model outperformed the two radiologists in terms of accuracy, and the DL model also demonstrated increased specificity. But the DL model exhibited reduced sensitivity. This finding may be explained by the fact that radiologists are more prone to suspect a tumor to be muscularly invasive due to their fear of the negative consequences of missing MIBC. Moreover, surprisingly, Reader 1, who had less experience in urogenital imaging, demonstrated better performance than Reader 2. Thus, a radiologist’s experience may not necessarily have a positive correlation with prediction accuracy. On the other hand, our results also indicated that the DL model could produce a more stable, objective and balanced outcome for discrimination between MIBC and NMIB compared to subjective assessment by radiologists.

ROI segmentation is an essential part of the research process. Currently, 3D segmentation of the whole tumor is widely adopted by researchers because it is thought to provide a more comprehensive evaluation compared to one ROI from the largest cross-sectional area of the tumor. Researchers typically need to manually draw the outline of the tumor on each image slice, which is time consuming, especially when the study population is large. Automated segmentation has been proposed, but the accuracy for BCa remains unclear. In this study, we applied a semiautomatic approach to segment each tumor. This method is a combination of automated segmentation by the platform and small modification by the radiologist. According to our experience, this semiautomatic method not only greatly accelerates the study process but also ensures the accuracy of ROI delineation. The interclass dice coefficient of 0.706 for ROI segmentation was slightly low. We analyzed those significantly different segmentations between the two radiologists and found that the radiologist with less experience mistakenly identified BCa lesions in patients with an irregular bladder shape or with prostate hyperplasia. This radiologist also failed to correctly segment some BCa lesions that presented as abnormal enhancement of the focal bladder wall. This result reminded us of the importance of the experience and the training of radiologists for ROI segmentation to reach a solid and reliable result.

Although the results were not very satisfactory, our study still has several strengths. First, this study explored the capacity of a DL model based on CT images to determine the status of muscle invasiveness of BCa, which provided a basis for subsequent studies to apply this technique to tackle relevant clinical problems. Second, unlike some other studies that used cross validation or single-center validation, this study used an external validation cohort enrolled from a different hospital, which allowed us to investigate the generalizability of the DL model. In addition, the study population of this study was larger than many other studies focusing on the application of machine learning in BCa. Third, CT-related studies of discriminating MIBC from NMIBC are limited. There is no doubt that CT has its limitations due to its low resolution of soft tissue. However, our study indicated that with the help of novel techniques, such as DL, we can also obtain valuable information from routine CT images to guide patient therapy. Thus, it is still worth performing CT-based studies to solve clinical problems in BCa management.

Our study has some limitations. First, this is a two-center study, but the number of patients in Center 2 is relatively small. Multicenter studies with larger population or prospective clinical trials should be conducted to validate the results in the future. Second, the proposed DL model exhibited its potential but the performance was less than satisfactory and has yet to be improved. Constant efforts should be made to optimize the model before it could be applied in real clinical practice. Third, the model was based on visible tumors on enhanced CT given that we excluded tumors detected by cystoscopy but invisible on CT images. Although these tumors constitute a small proportion of BCa, they may still limit the scope of the model’s application to some extent. Fourth, in this study, we did not incorporate other clinical information which may be helpful for determining the invasiveness of BCa, such as urine DNA or RNA. We aimed to investigate the potential of deep learning to facilitate CT evaluation of BCa, thus we focused on CT images only. It’s possible that integrating those useful clinical information into the model might further improve the prediction accuracy. Fifth, we only chose the largest one among multiple lesions for segmentation and there is a chance that the largest one didn’t have the highest T stage. But usually larger lesions are supposed to have higher T stage, and it’s very difficult to make one-to-one correspondence between the lesion on CT images and the lesion pathologist evaluated, we think choosing the largest one for analysis is acceptable.

In conclusion, we developed a DL model based on enhanced CT images to predict muscle invasiveness of BCa. This model should favorable performance. It could provide more useful information for individual preoperative evaluation, may facilitate clinical decision making and improve patient care.

## Data Availability Statement

The original contributions presented in the study are included in the article/**Supplementary Material**. Further inquiries can be directed to the corresponding author.

## Ethics Statement

The studies involving human participants were reviewed and approved by the Institutional Review Board of Peking Union Medical College Hospital and Fushun Central Hospital of Liaoning Province. Written informed consent for participation was not required for this study in accordance with the national legislation and the institutional requirements.

## Author Contributions

GZ: conception and design, acquisition of data, analysis and interpretation of data, manuscript drafting and revision, and statistical analysis. ZW: acquisition of data, manuscript drafting and revision, and administrative and material support. LX: acquisition of data. XZ: analysis and interpretation of data. DZ: analysis and interpretation of data. YX: acquisition of data. LM: analysis and interpretation of data, and statistical analysis. XL: conception and design, manuscript drafting and revision, and administrative and material support. JG: acquisition of data. ZhiJ: administrative and material support. HS: conception and design, manuscript drafting and revision, administrative and material support, and supervision. ZheJ: administrative and material support, and supervision. All authors contributed to the article and approved the submitted version.

## Funding

This work was supported by the National Natural Science Foundation of China [81901742, 91859119], the Natural Science Foundation of Beijing Municipality [7192176], the Clinical and Translational Research Project of Chinese Academy of Medical Sciences [XK320028], and the National Public Welfare Basic Scientific Research Project of Chinese Academy of Medical Sciences [2018PT32003, 2019PT320008].

## Conflict of Interest

LM and XL are employees of Deepwise AI Lab, Deepwise Inc., which contributed to the development of radiomics models described in the study.

The remaining authors declare that the research was conducted in the absence of any commercial or financial relationships that could be construed as a potential conflict of interest.
