# Supplementary material for: Deep Learning on Enhanced CT Images Can Predict the Muscular Invasiveness of Bladder Cancer
Source: Front Oncol. 2021 Jun 11;11:654685. doi: 10.3389/fonc.2021.654685 (PMC8226179; doi:10.3389/fonc.2021.654685)
Supplement: Supplementary file 1 [file Table_1.docx]

**Supplementary Table S1. The CT acquisition parameters of the two centers.**

|  | **Parameters** | **Center 1** | | | **Center 2** |
| --- | --- | --- | --- | --- | --- |
| **CT system**  **information** | CT system | Dual Source CT (Somatom Definition Flash, Siemens Healthcare, Germany) | | Spectral CT (Discovery CT750 HD scanner, GE Medical Systems, USA) | 64-channel CT (Brilliance CT, ROYAL PHILIPS, Netherlands) |
| **CT scan parameters** | Tube voltage | 120 kVp | | 120kVp | 120kVp |
|  | Tube current | Variable tube current  with automatic tube-current modulation activated | | Variable tube current  with automatic tube-current modulation activated | 300mAs |
|  | Rotation time | 0.5s | | 0.6 s | 0.75s |
|  | Detector collimation | 64×0.6 mm | | 64×0.625 mm | 64×0.625 mm |
|  | Pitch | 0.9 | | 0.9 | 0.9 |
|  | Nephrographic phase | 80s after injection | 80s after injection | | 80s after injection |
|  | Image matrix | 512×512 | 512×512 | | 512×512 |
|  | Field of view | 350×350 mm | 350×350 mm | | 350×350 mm |
|  | Reconstruction section thickness | 1mm | 1mm | | 1mm |
